# COVID-19 risk perceptions after the end of the public health emergency

**DOI:** 10.1371/journal.pone.0348400

**Published:** 2026-11-10

**Authors:** Asako Chiba, Kazuya Haganuma, Taisuke Nakata, Thuy Linh Nguyen, Reo Takaku

**Affiliations:** 1 Department of Economics, Keio University, Minato, Tokyo, Japan; 2 School of Veterinary Science, College of Sciences, Massey University, Palmerston North, New Zealand; 3 Faculty of Economics and Graduate School of Public Policy, University of Tokyo, Bunkyo, Tokyo, Japan; 4 Faculty of Economics, University of Tokyo, Bunkyo, Tokyo, Japan; 5 Graduate School of Economics, Hitotsubashi University, Kunitachi, Tokyo, Japan; Takushoku University, JAPAN

## Abstract

We examine how information provision affects the public’s perceived COVID-19 infection risk after the official end of the pandemic as a public health emergency (PHE). We conducted our survey in Japan in August 2023, a few months after the government reclassified COVID-19 from Category II to Category V and officially ended the PHE. We find that none of the information treatments affected the public’s risk perceptions in a statistically significant way, in stark contrast with a similar information-provision experiment conducted right before the reclassification. Our results are consistent with reduced public responsiveness to news about infection following the official end of the PHE.

## Introduction

Throughout the COVID-19 pandemic, the public was exposed to various types of information about infection risk, ranging from official statistics and policy announcements to expert commentary and media reports. Such information likely influenced how people perceived the likelihood and consequences of infection and thereby affected preventive behaviors. Accordingly, a better understanding of risk perceptions can give us insights into behavioral responses during public-health crises and help policymakers design effective public communication strategies in a future pandemic.

In this study, we examine how information provision affects the public’s perceived COVID-19 infection risk after the official end of the pandemic as a public health emergency (PHE). We fielded a survey experiment in Japan between August 14 and August 28, 2023 (the orange shaded area in Fig 1), a few months after the government’s reclassification of COVID-19 from Category II to Category V on May 8, 2023. The reclassification marked the end of the PHE. We randomized respondents into four treatment groups, each receiving one of four real news items published in Japan in July 2023, or into a control group that received no additional information. All four information treatments are qualitative, as opposed to quantitative. Three of them provided a pessimistic infection outlook, whereas one of them provided an optimistic outlook.

**Fig 1:**
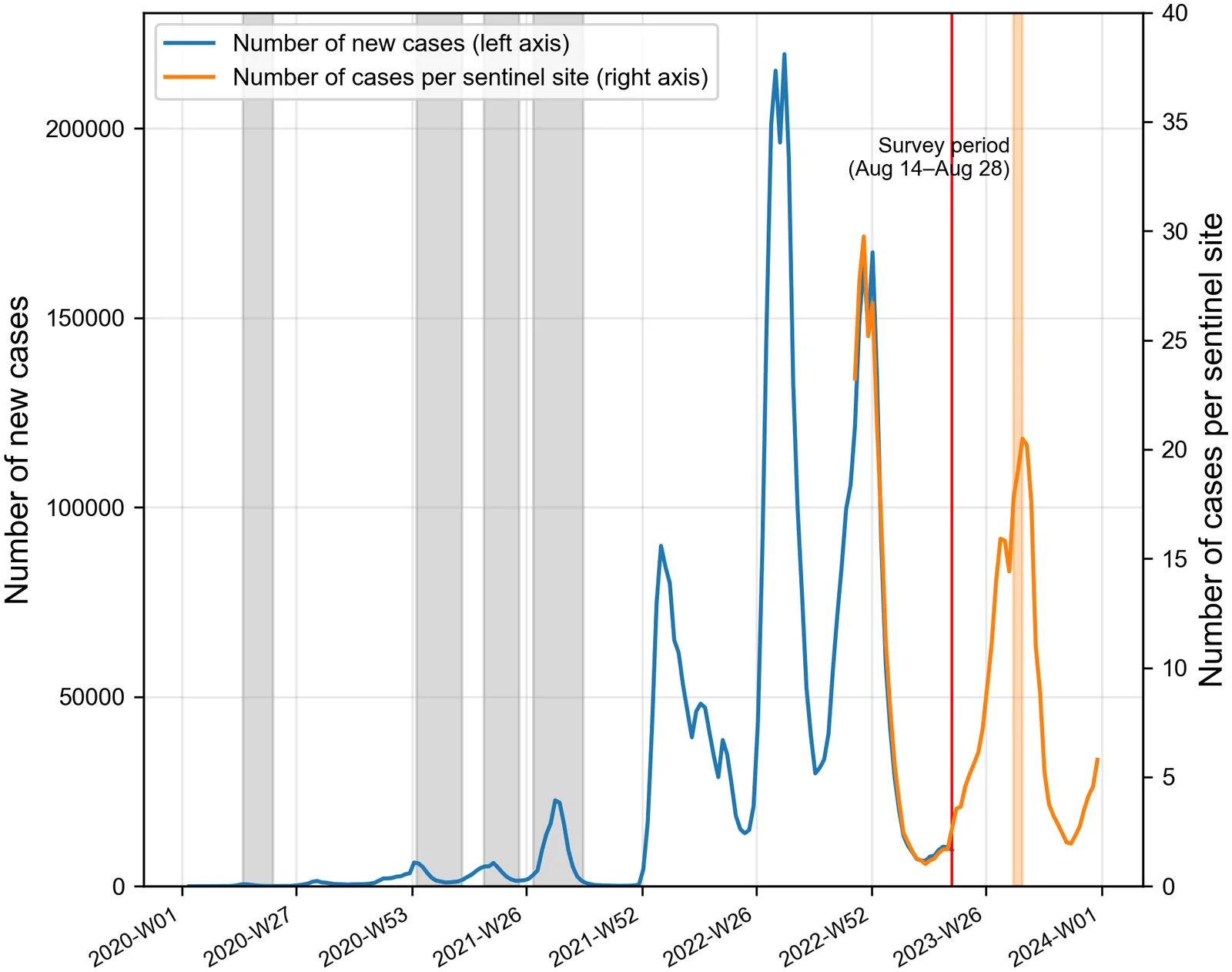
COVID-19 infections and the timing of our survey. This figure plots the number of new COVID-19 infections in Japan. The blue line (left y-axis) shows the weekly average of daily new infections from 2020 through May 2023. Following the reclassification of COVID-19 from Category II to Category V on May 8, 2023 (red vertical line), nationwide case counts were replaced by sentinel surveillance (Teiten Kansoku), under which designated medical institutions report new infections. The orange line (right y-axis) shows the weekly number of cases per sentinel site from May 2023 to December 2023. Grey shaded areas indicate periods with a state-of-emergency order in Tokyo, and the orange shaded area marks the survey period (August 14–28, 2023). Data are from the Ministry of Health, Labour and Welfare [1,2] and the Japan Institute for Health Security [3].

A better understanding of public risk perceptions is important even after the official end of the pandemic as a public health emergency for the following reason. In some countries, normalization from the COVID-19 crisis was slow and prolonged. For example, [4] show that even after the pandemic ends, many planned to continue reducing in-person interactions for an extended period. More broadly, the literature documents persistent pandemic effects across a range of outcomes, including sectoral reallocation and labor-market churn [5], distributional impacts and labor-market scarring that disproportionately affect lower-wage workers [6], scarring from disruptions to children’s human capital accumulation [7], and longer-lasting mental-health consequences [8]. Because perceived infection risk likely influences precautionary behavior—and therefore social and economic activity—how people perceive the infection risk after the official end of COVID-19 is likely to shape the pace of normalization.

We emphasize two main findings. First, on average, none of the information treatments meaningfully changed respondents’ perceived infection risk. This finding is robust to alternative experimental setups and is common across major demographic subgroups. This finding is in stark contrast to [9] which conducted a similar experiment in April 2023 and found quantitatively large and statistically significant effects of pessimistic, qualitative information on risk perceptions. Second, though we detect some statistically significant heterogeneous responses for a small number of subgroups, such as respondents with high prior risk perceptions or chronic conditions, these effects are rare and generally not robust across alternative setups.

The contrasting findings between this study and [9] have important policy implications. Because the two surveys are separated by only a few months, the difference in the findings suggests that the responsiveness of risk beliefs to information can change quickly when the institutional environment changes. A natural explanation is that Japan’s widely publicized May 2023 reclassification—the official end of the pandemic as a PHE—served as a salient public signal that reset baseline beliefs and reduced the marginal informational content of subsequent COVID-related news.

Our study contributes to three strands of the literature. First, our paper is related to a set of papers investigating the public’s subjective assessments of COVID-19 risks. One line of research compares perceived COVID-19 risks to objective benchmarks [10,11]. Another line of research studies how subjective risk beliefs map into preventive behaviors [12–16]. Others examine factors associated with subjective risk [17]. We differ from this literature in design: Rather than relying primarily on correlational evidence, we implement a randomized information-provision experiment to identify the effect of information exposure causally.

Within this first strand of literature, our paper is most closely related to the analysis of COVID-19 risk perceptions in Japan. See, for example [18–25]. None of these papers—except [26]—conduct information-provision experiments, whereas we do. All papers—except for [19]—conduct their surveys in 2020 and 2021, well before the official end of the PHE in 2023. Our paper is unique because we conduct an information-provision experiment in the summer of 2023, after the reclassification of COVID-19 to Category V.

Second, our work is closely related to information-provision experiments in the COVID-19 context. A large experimental literature studies how information affects preventive behaviors, including vaccination intentions [27–30], social distancing [31,32], mask wearing [33–35] and other health-protective actions. In contrast to this behavior-focused literature, we study the effect of information provision on people’s COVID-19 risk perception.

Third, our paper contributes to the intersection of the above two strands: information treatments that directly target subjective COVID-19 risk perceptions. Existing experiments that link information provision to belief updating about COVID-19 risk provide important evidence during the acute phase of the pandemic [36–39]. We differ from this literature by focusing on a dimension that has received limited attention: the post-reclassification period.

The structure of the paper is as follows. Section Methods describes the survey and experimental design. Section Results presents our main results. Section Discussion discusses the implications of our findings. Section Conclusion concludes.

## Methods

### Setting and respondents

We conducted a nationally representative online survey of Japanese residents in collaboration with Cross Marketing, Inc., an online marketing firm. The survey was conducted between August 14 and August 28, 2023. This survey period was a few months after the official end of Japan’s COVID-19 pandemic response (following the May 2023 reclassification) and around the peak of the ninth infection wave. The survey targeted men and women aged 20 years and older, yielding a valid sample size of 5,000. The survey asked respondents about their perceived risk of COVID-19 infection as well as a range of individual characteristics. To ensure the representativeness of our sample, the proportions of gender (Male, Female) and age cohort (20s-30s, 40s-50s, ≥60s) were stratified to match the demographic distribution reported in the 2020 Population Census. We did not stratify our sample according to respondents’ location of residence. Nevertheless, our sample does include respondents from all 47 prefectures of Japan, covering both densely and sparsely populated areas.

This survey experiment is registered in the AEA RCT Registry as AEARCTR-0017970. We obtained ethics approval from the Ethics Review Board at the University of Tokyo (Approval No. 23–221). All procedures adhered to the relevant guidelines and regulations. Informed consent was obtained electronically from all participants prior to participation through the online survey. The full questionnaire is provided in Supplementary Information S1 Appendix.

### Prior to information provision

Our goal was to examine whether providing pessimistic, qualitative information about COVID-19 risk affects individuals’ risk perceptions. We first elicited each respondent’s prior risk perceptions before any experimental information was provided. Specifically, we elicited the subjective probability of being infected with COVID-19 over the next one month by presenting the following response options to the respondents: (1) less than 0.001%, (2) 0.001% to less than 0.01%, (3) 0.01% to less than 0.1%, (4) 0.1% to less than 1%, (5) 1% to less than 5%, (6) 5% to less than 10%, (7) 10% to less than 20%, and (8) 20% or higher.

Before we elicited these subjective probabilities, we presented two numerical reference points as nominal anchors: (1) the realized infection risk as of July 2022, when infections were spreading rapidly (2.77%), and (2) the realized infection risk as of October 2022, when the spread had slowed (0.83%). Anchors are held constant across arms and are used to reduce extreme entries and improve measurement precision rather than to shift beliefs differentially.

### Information provision

When providing information about COVID-19, we divided respondents into five groups (each consisting of 1,000 respondents) and randomly assigned respondents to these groups. With 1,000 respondents per group, a power analysis based on a two-sample t-test (α = 0.05, power = 0.8) indicates a minimum detectable effect of 0.125 standard deviations. This suggests that the sample size is sufficient to detect economically meaningful effects.

For all five groups, we first presented the observed infection risk as of April 2023, near the end of the pandemic (0.21%). We then provided additional COVID-related information that varied by group: one group received no additional information (control group); one group received a comment by a clinic in Tokyo warning about the potential collapse of the medical system; one group received a statement from a hospital in Okinawa made at a press conference, also warning about the potential collapse of the medical system; one group received a comment by a COVID-19 expert stating that the spread of infection will likely continue; and one group received a statement by a government official indicating that Japan is currently not in the middle of the ninth infection wave. The exact wording of the information provided is as follows:

#### Clinic in Tokyo

*‘In early July, the director of a clinic in Tokyo said that “the number of patients is on par with the peak of the eighth wave (the number of infections exceeded 20,000 cases per day in Tokyo), and an invisible collapse of the medical system has begun.” ’*

#### Hospital in Okinawa

*‘A hospital in Okinawa held an emergency press conference on July 11 to announce the risk of collapse of the medical system due to a resurgence of infections, urging citizens not to relax basic preventive measures.’*

#### Expert communication

*‘On July 16, Shigeru Omi, the head of the government’s COVID-19 countermeasures subcommittee, stated that “not only the number of new cases but also the number of hospitalized and severe cases are increasing. This could be due to an increase in contact opportunities following the reclassification of COVID-19 and to diminished immunity from natural infections and vaccines over time. We do not know the full extent of the increase in the number of infections, but we should anticipate that this upward trend will continue.” ’*

#### Government information

*‘At a press conference held in early July, Shigeyuki Goto, the minister in charge of measures for novel coronavirus disease, stated that the number of patients had not increased sharply, and it was inappropriate to consider the current period as the ninth wave of infections in Japan.’*

We interpret the first three information treatments as pessimistic and the last information treatment as optimistic.

To assess the extent of success in randomization, we examine whether the randomization actually generated comparable groups by conducting balance tests on pre-treatment characteristics. Table 1 shows the result. The first five columns report group-specific means for each characteristic, and the remaining columns present p-values from two-sided t-tests that compare each treatment group to the control group. In nearly all cases, the p-values exceed 0.1, indicating that observable characteristics are well balanced across groups. A key exception is “acquaintance died from COVID-19.” According to the last row of the table, the proportion is lower in Treatment Groups (Tokyo and Okinawa) than in the control group in a statistically significant way. This imbalance is unlikely to be relevant in most of our regression analyses because we do control for this characteristic (see Section Results). However, in the analysis examining the heterogeneity in terms of “acquaintance died from COVID-19,” we cannot completely rule out the possibility that this imbalance influences the estimated treatment effects.

**Table 1:** Balance test.

	*Mean values*					*P-values (t-test*)			
	No Info.	Tokyo	Okinawa	Expert	Gov.	Tokyo	Okinawa	Expert	Gov.
Age	50.410	50.482	50.422	50.300	50.419	0.918	0.986	0.875	0.990
Female	0.505	0.505	0.505	0.505	0.505	1.000	1.000	1.000	1.000
College Graduate	0.483	0.484	0.485	0.471	0.485	0.964	0.929	0.591	0.929
High Income	0.505	0.521	0.520	0.497	0.497	0.474	0.502	0.721	0.721
Vaccinated	0.851	0.853	0.851	0.834	0.848	0.900	1.000	0.297	0.851
Chronic Diseases	0.134	0.143	0.146	0.139	0.142	0.560	0.440	0.745	0.604
Infected with COVID-19	0.246	0.240	0.243	0.225	0.248	0.755	0.876	0.269	0.918
Acq. Died from COVID-19	0.085	0.053	0.065	0.068	0.083	0.005	0.090	0.153	0.872

*Notes*: This table presents the results of covariate balance tests. The first five columns report mean values of each covariate for the control group and the four treatment groups. The last four columns report p-values from two-sided t-tests comparing each treatment group to the control group. ‘Acq.’ denotes acquaintance.

### Post information provision

After the information provision, we elicited respondents’ posterior risk perceptions by asking them again about their subjective probability of becoming infected with COVID-19 within the next one month. We used the same question format and response categories as in the pre-information stage, which allows a direct comparison of beliefs before and after exposure to the assigned information.

At this stage, we also collected respondents’ background information, including education, household income in 2022, health conditions, vaccination status (number of vaccine doses), COVID-19 related experiences (the number of times infected and whether the respondent used ECMO/ventilator support), whether any acquaintances died from COVID-19, and the extent of engagement in infection-prevention behaviors over the past month as well as intended engagement over the next month.

Detailed summary statistics regarding individual attributes are presented in Table 2. The respondents’ age and gender are distributed closely to those of residents in Japan by construction. The distribution of educational attainment shows a somewhat larger share of college graduates than in official statistics: It is 48.2 percent in our survey, whereas it is 25.7 percent according to the National Census. Income distribution is close to that in the official statistics: 50.8 percent earned more than 4 million yen in our survey, whereas 49.4 percent earned more than 4 million according to the Statistical Survey of Actual Status for Salary in the Private Sector in 2023. Overall, these data suggest that the respondents represent residents in Japan reasonably well.

**Table 2:** Summary statistics.

	Total
*Age*	
20-59 years	3,285 (65.7%)
Over 60 years	1,715 (34.3%)
*Gender*	
Male	2,475 (49.5%)
Female	2,525 (50.5%)
*Education Level*	
Non-College Graduate	2,592 (51.8%)
College Graduate	2,408 (48.2%)
*Income (in ten-thousand yen)*	
Less than 400	2,460 (49.2%)
400 or more	2,540 (50.8%)
*Vaccination*	
Unvaccinated	763 (15.3%)
Vaccinated	4,237 (84.7%)
*Chronic Diseases*	
Yes	704 (14.1%)
No	4,296 (85.9%)
*Infected with COVID-19*	
Yes	1,202 (24.0%)
No	3,798 (76.0%)
*Acq. Died from COVID-19*	
Yes	354 (7.1%)
No	4,646 (92.9%)
**N**	5,000

*Notes*: This table reports summary statistics for respondents’ individual attributes. Numbers denote counts, and percentages are in parentheses.

### Two alternative setups

For robustness, we conduct the same information-provision experiment described thus far in two slightly modified setups. The first alternative setup is identical to the baseline setup, except that we do not provide our respondents with nominal anchors before eliciting the prior subjective risk perceptions. This allows us to rule out the possibility that nominal anchors substantially shift beliefs and affect our treatment effects.

The second alternative setup is identical to the first alternative setup, except that we do not elicit prior subjective risk perceptions. This allows us to rule out experimenter-demand effects from eliciting priors—that is, respondents may infer the importance of risk perceptions and adjust their subsequent responses. The key results from the baseline setup described in the following section are robust to these two alternative setups. The robustness results are discussed in the Supplementary Information S2 Appendix.

## Results

### Risk perceptions prior to information provision

We begin by describing respondents’ risk perceptions prior to information provision. Fig 2 plots the distribution of subjective infection risk. The blue vertical lines indicate summary statistics of subjective beliefs (the sample mean and median), while the red vertical line indicates the realized infection risk during the one-month period from the survey start date (August 14 – September 13, 2023).

**Fig 2:**
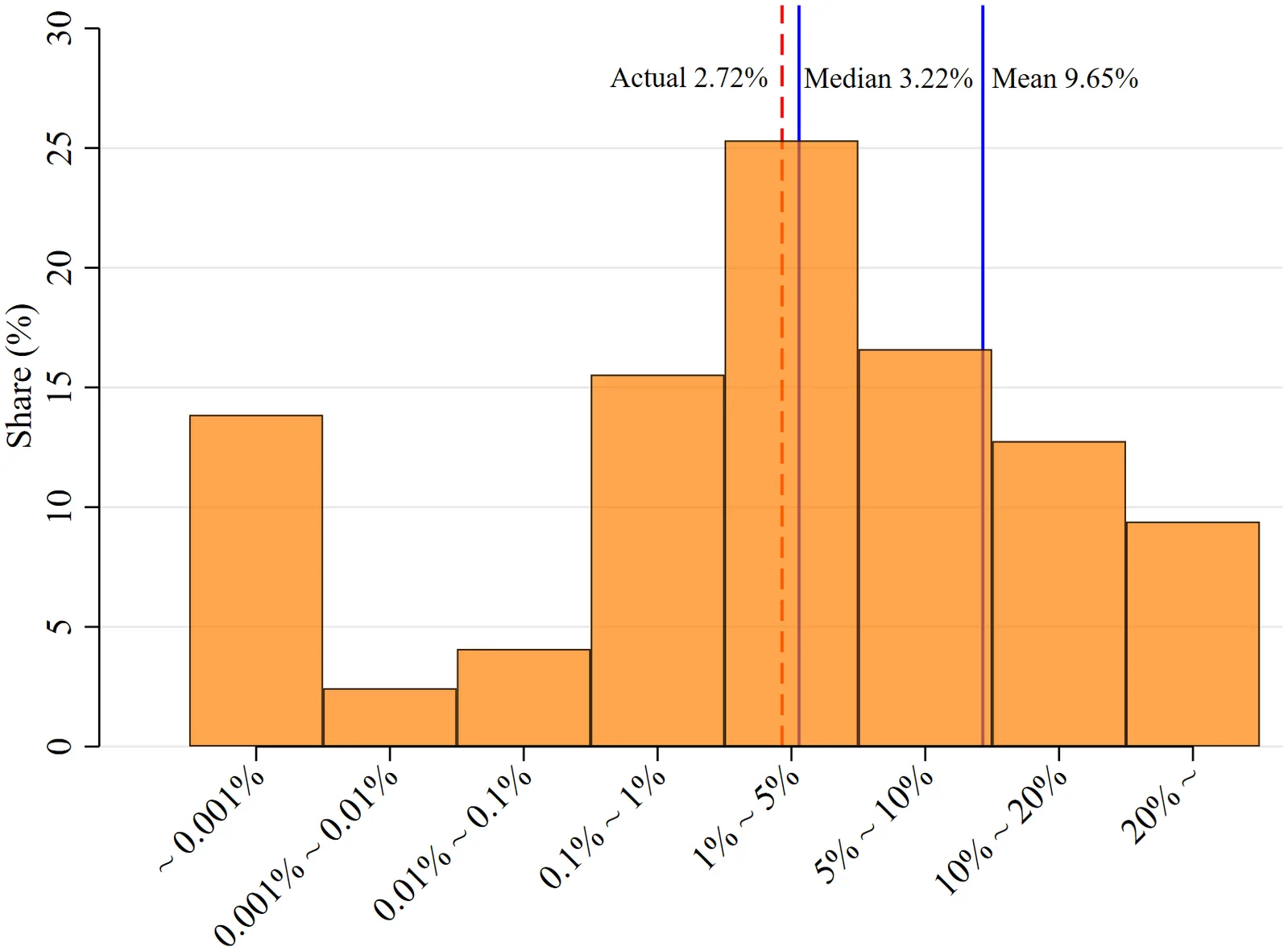
Distribution of prior risk perceptions. This figure shows the distribution of subjective infection risk. The blue vertical lines indicate the mean and median of perceived risk, while the red vertical line indicates the realized infection risk. The realized risk is estimated by combining two COVID-19 infection series shown in Fig 1. Specifically, we regress weekly average number of new infections (left y-axis) on cases per sentinel site (right y-axis) using data from December 2022 to May 2023. We then apply the estimated coefficient to sentinel-site data to compute weekly average number of new infections during August 14 – September 13, 2023. We aggregate these estimates and divide the total infections by the total population [40] to obtain the realized risk.

The figure shows substantial risk overestimation relative to the realized risk: A large mass of respondents reports infection probabilities far above the realized risk of 2.72%. In particular, 38.8% (16.6 + 12.8 + 9.4) respondents believed that their likelihood of contracting COVID-19 within the next month was 5% or greater. Setting a higher threshold, we find that 22.2% (12.8 + 9.4) of the respondents assessed the infection risk as 10% or higher. At the same time, a non-negligible share understates the risk: 13.9% report that their infection risk is almost zero (less than 0.001%).

Overall, the distribution is wide and centered well above the realized risk, consistent with our earlier surveys on COVID-19 risk perceptions in Japan [9,41].

### Effects of information provision

To examine the effects of information provision on reported infection risk, we estimate the following regression specification:


$$\begin{array}{c} y_{i}=\alpha +\sum_{j=1}^{4}\beta_{j}D_{ji}+{𝐱}_{i}^{\prime }\gamma +\epsilon_{i} \end{array}$$ (1)

where the dependent variable ($y_{i}$) is subjective infection risk, defined as the midpoints of each response category. Note that for open-ended categories of 20% or higher and less than 0.001%, we top-code at 100% and bottom-code at 0%, assigning midpoints of 60% and 0.0005%, respectively. The independent variables (*D*_1*i*_,*D*_2*i*_,*D*_3*i*_,*D*_4*i*_) are dummy variables for ‘Tokyo’, ‘Okinawa’, ‘Expert’ and ‘Government’ information group, respectively. The set of control variables (${𝐱}_{i}$) include dummy variables for high prior risk perceptions (prior risk ≥10%), age, gender, education, income, chronic diseases, infection history (0 or ≥1), acquaintance’s COVID-19-related deaths, and vaccination status. Although treatment assignment was randomized, we include these control variables to account for variation in risk perceptions associated with individual characteristics, thereby improving the precision of the estimated treatment effects. We also control for prefecture fixed effects and for the primary type of media respondents used to obtain information about COVID-19, considering that respondents were under different measures and situations of COVID-19 across prefectures and under different accuracy and accessibility to information across media. However, in all tables presenting regression results, the coefficients on media types and prefectures are omitted for simplicity.

Table 3 reports the estimation results. All analyses were conducted using Stata (version 17). Two-sided *p*-values below 0.05 are treated as statistically significant. For each group dummy variable included in the regression, if the estimated coefficient is positive (negative), respondents in that group perceive the risk to be higher (or lower) than those in the control group. According to the table, all treatment coefficients are small in magnitude and not statistically significantly different from zero. This result is robust to alternative experimental setups and remains qualitatively similar across demographic subgroups.

**Table 3:** Average effects.

	(1) Infection Risk
Information: Tokyo	0.453
	(0.554)
Information: Okinawa	−0.331
	(0.544)
Information: Expert	0.434
	(0.557)
Information: Government	−0.629
	(0.533)
Prior Risk Above 10%	24.303***
	(0.712)
College Graduate	−0.109
	(0.367)
High Income	−0.155
	(0.361)
Age Over 60 years	−0.703*
	(0.375)
Female	0.640*
	(0.360)
Vaccination	0.263
	(0.491)
Chronic Diseases	0.614
	(0.507)
Infected with COVID-19	0.114
	(0.443)
Acq. Died from COVID-19	0.528
	(0.787)
Constant	3.358***
	(1.021)
Observations	5,000
R-squared	0.416

*Notes*: This table reports regression results examining the average treatment effects of information provision. The dependent variable is subjective infection risk. The independent variables are dummy variables for information groups. Control variables include dummy variables for prior risk perception, age, gender, education, income, chronic diseases, prior infection, acquaintance’s COVID-19-related death, and vaccination status. All regressions also include prefecture fixed effects and dummy variables for primary media type; however, coefficients for these variables are omitted from the table for brevity. Robust standard errors are in parentheses. * p < 0.1, ** p < 0.05, *** p < 0.01.

We now turn to the analysis of heterogeneous treatment effects. For this analysis, we partition the sample by respondents’ demographic and socioeconomic characteristics as well as their COVID-19 experiences, and estimate the following equation:


$$\begin{array}{c} y_{i}=\alpha +\delta H_{i}+\beta D_{i}+\eta D_{i}\times H_{i}+{𝐱}_{i}^{\prime }\gamma +\epsilon_{i} \end{array}$$ (2)

For this analysis, we added to our baseline regressions in (1) (presented in Table 3) the interaction terms between the information group dummy variables and each characteristic of interest ($H_{i}$)—prior risk perceptions, age, gender, education, income, health condition, COVID-19-related experiences, and vaccination status. Across a wide range of characteristics, the interaction coefficients are generally small and statistically insignificant, suggesting that most observable characteristics are not associated with systematically different responses to the information treatments.

Nevertheless, we detect statistically significant responses for a small number of subgroups in some specifications. Table 4 shows that, for respondents with high prior risk perceptions (prior risk ≥10%), the Okinawa treatment is associated with a negative and statistically significant interaction at the 5% level, and the Government treatment yields a negative interaction that is marginally significant at the 10% level. Among respondents with chronic diseases (see Table 5), the Okinawa treatment generates a negative interaction significant at the 5% level, while the Expert treatment produces a positive interaction that is marginally significant at the 10% level. We also find a few additional isolated significant interactions. For example, the Expert treatment interacted with age 60 + is positive and significant at the 5% level, and the Government treatment interacted with being female or having an acquaintance who died from COVID-19 is negative and marginally significant at the 10% level (see Tables 6–8).

**Table 4:** Heterogeneity – Prior risk.

	(1)	(2)	(3)	(4)
GROUP	Control & Tokyo	Control & Okinawa	Control & Expert	Control & Gov.
VARIABLES	Posterior Risk	Posterior Risk	Posterior Risk	Posterior Risk
Prior Risk Above 10%	26.130***	26.050***	26.182***	26.123***
	(1.512)	(1.516)	(1.506)	(1.513)
Treatment*Prior Risk Above 10%	−0.655	−5.402**	0.245	−3.842*
	(2.246)	(2.205)	(2.183)	(2.145)
Treatment dummy	0.579*	0.857**	0.287	0.229
	(0.313)	(0.333)	(0.301)	(0.255)
Observations	2,000	2,000	2,000	2,000
R-squared	0.457	0.426	0.472	0.453

*Notes*: This table reports regression results examining heterogeneous treatment effects by prior infection risk. The dependent variable is subjective infection risk. The independent variables are *Prior Risk Above 10%*, the treatment group dummy, and their interaction. All specifications control for individual characteristics, prefecture fixed effects, and primary media type dummies. However, coefficients for these variables are omitted from the table for brevity. In each column, the regression sample consists of respondents in the control group and one treatment group—Tokyo, Okinawa, Expert, and Government, respectively. Robust standard errors are in parentheses. * p < 0.1, ** p < 0.05, *** p < 0.01.

**Table 5:** Heterogeneity – Health.

	(1)	(2)	(3)	(4)
GROUP	Control & Tokyo	Control & Okinawa	Control & Expert	Control & Gov.
VARIABLES	Posterior Risk	Posterior Risk	Posterior Risk	Posterior Risk
Chronic Diseases	1.256	0.946	0.791	0.999
	(1.170)	(1.150)	(1.166)	(1.156)
Treatment*Chronic Diseases	−0.833	−3.149**	3.041*	−1.009
	(1.622)	(1.510)	(1.707)	(1.597)
Treatment dummy	0.548	0.126	−0.070	−0.522
	(0.608)	(0.611)	(0.592)	(0.580)
Observations	2,000	2,000	2,000	2,000
R-squared	0.457	0.422	0.473	0.450

*Notes*: This table reports regression results examining heterogeneous treatment effects by health status. The dependent variable is subjective infection risk. The independent variables are *Chronic Diseases*, the treatment group dummy, and their interaction. All specifications control for individual characteristics, prefecture fixed effects, and primary media type dummies. However, coefficients for these variables are omitted from the table for brevity. In each column, the regression sample consists of respondents in the control group and one treatment group—Tokyo, Okinawa, Expert, and Government, respectively. Robust standard errors are in parentheses. * p < 0.1, ** p < 0.05, *** p < 0.01.

**Table 6:** Heterogeneity – Age.

	(1)	(2)	(3)	(4)
GROUP	Control & Tokyo	Control & Okinawa	Control & Expert	Control & Gov.
VARIABLES	Posterior Risk	Posterior Risk	Posterior Risk	Posterior Risk
Age Over 60 years	−1.814**	−1.890**	−2.041***	−1.821**
	(0.779)	(0.772)	(0.780)	(0.774)
Treatment*Age Over 60 years	0.305	1.024	2.835**	0.766
	(1.098)	(1.102)	(1.125)	(1.090)
Treatment dummy	0.325	−0.668	−0.636	−0.923
	(0.732)	(0.706)	(0.729)	(0.718)
Observations	2,000	2,000	2,000	2,000
R-squared	0.457	0.421	0.473	0.450

*Notes*: This table reports regression results examining heterogeneous treatment effects by age. The dependent variable is subjective infection risk. The independent variables are *Age Over 60 years*, the treatment group dummy, and their interaction. All specifications control for individual characteristics, prefecture fixed effects, and primary media type dummies. However, coefficients for these variables are omitted from the table for brevity. In each column, the regression sample consists of respondents in the control group and one treatment group—Tokyo, Okinawa, Expert, and Government, respectively. Robust standard errors are in parentheses. * p < 0.1, ** p < 0.05, *** p < 0.01.

**Table 7:** Heterogeneity – Gender.

	(1)	(2)	(3)	(4)
GROUP	Control & Tokyo	Control & Okinawa	Control & Expert	Control & Gov.
VARIABLES	Posterior Risk	Posterior Risk	Posterior Risk	Posterior Risk
Female	0.979	0.689	1.082	1.008
	(0.794)	(0.802)	(0.797)	(0.799)
Treatment*Female	−0.674	0.182	0.658	−1.844*
	(1.139)	(1.092)	(1.130)	(1.092)
Treatment dummy	0.769	−0.409	0.011	0.271
	(0.754)	(0.739)	(0.740)	(0.714)
Observations	2,000	2,000	2,000	2,000
R-squared	0.457	0.421	0.472	0.451

*Notes*: This table reports regression results examining heterogeneous treatment effects by gender. The dependent variable is subjective infection risk. The independent variables are *Female*, the treatment group dummy, and their interaction. All specifications control for individual characteristics, prefecture fixed effects, and primary media type dummies. However, coefficients for these variables are omitted from the table for brevity. In each column, the regression sample consists of respondents in the control group and one treatment group—Tokyo, Okinawa, Expert, and Government, respectively. Robust standard errors are in parentheses. * p < 0.1, ** p < 0.05, *** p < 0.01.

**Table 8:** Heterogeneity – Acquaintance death.

	(1)	(2)	(3)	(4)
GROUP	Control & Tokyo	Control & Okinawa	Control & Expert	Control & Gov.
VARIABLES	Posterior Risk	Posterior Risk	Posterior Risk	Posterior Risk
Acq. Died from COVID-19	1.602	1.568	1.607	1.325
	(1.386)	(1.402)	(1.362)	(1.384)
Treatment*Acq. Died from COVID-19	−3.430	2.260	−0.556	−3.874*
	(2.240)	(2.455)	(2.508)	(2.015)
Treatment dummy	0.655	−0.482	0.386	−0.339
	(0.578)	(0.559)	(0.570)	(0.557)
Observations	2,000	2,000	2,000	2,000
R-squared	0.457	0.421	0.472	0.451

*Notes*: This table reports regression results examining heterogeneous treatment effects by COVID-19-related acquaintance death. The dependent variable is subjective infection risk. The independent variables are *Acq. Died from COVID-19*, the treatment group dummy, and their interaction. All specifications control for individual characteristics, prefecture fixed effects, and primary media type dummies. However, coefficients for these variables are omitted from the table for brevity. In each column, the regression sample consists of respondents in the control group and one treatment group—Tokyo, Okinawa, Expert, and Government, respectively. Robust standard errors are in parentheses. * p < 0.1, ** p < 0.05, *** p < 0.01.

Tables 9 and 10 examine how the effect of information treatment depends on population density and sources of information. For population density, we classify respondents based on whether they reside in a prefecture with above-median population density. For sources of information, we classify respondents based on whether their primary media source is traditional media (television or newspapers). According to Table 9, among respondents who reside in densely populated areas, the Expert and Government treatments generate positive interactions significant at least at the 10% level. According to Table 10, among respondents who mainly rely on traditional media, the Government treatment generates a negative interaction significant at the 5% level. We also conducted heterogeneity analyses along the dimensions of education level, income, vaccination status, and past COVID-19 infection experiences. For these four dimensions, we do not find statistically significant results for any of the information treatments (see S3 Appendix).

**Table 9:** Heterogeneity – Population density.

	(1)	(2)	(3)	(4)
GROUP	Control & Tokyo	Control & Okinawa	Control & Expert	Control & Gov.
VARIABLES	Posterior Risk	Posterior Risk	Posterior Risk	Posterior Risk
High Density	5.914	2.804	0.798	3.397
	(4.062)	(3.532)	(3.784)	(3.610)
Treatment*High Density	1.349	−0.673	2.837**	2.202*
	(1.385)	(1.422)	(1.418)	(1.294)
Treatment dummy	−0.653	0.214	−1.922	−2.407**
	(1.236)	(1.287)	(1.280)	(1.140)
Observations	2,000	2,000	2,000	2,000
R-squared	0.457	0.421	0.473	0.451

*Notes*: This table reports regression results examining heterogeneous treatment effects by population density. The dependent variable is subjective infection risk. The independent variables are *High Density* (a dummy variable equal to one if the respondent resides in a prefecture with above-median population density, and zero otherwise), the treatment group dummy, and their interaction. Prefecture-level population density used in this analysis is obtained from the 2020 Population Census. All specifications control for individual characteristics, prefecture fixed effects, and primary media type dummies. However, coefficients for these variables are omitted from the table for brevity. In each column, the regression sample consists of respondents in the control group and one treatment group—Tokyo, Okinawa, Expert, and Government, respectively. Robust standard errors are in parentheses. * p < 0.1, ** p < 0.05, *** p < 0.01.

**Table 10:** Heterogeneity – Source of information.

	(1)	(2)	(3)	(4)
GROUP	Control & Tokyo	Control & Okinawa	Control & Expert	Control & Gov.
VARIABLES	Posterior Risk	Posterior Risk	Posterior Risk	Posterior Risk
Traditional Media	1.081	1.902	4.017**	3.492
	(2.176)	(2.347)	(1.980)	(2.355)
Treatment*Traditional Media	−1.496	−1.803	0.635	−2.167**
	(1.128)	(1.111)	(1.119)	(1.097)
Treatment dummy	1.263	0.695	−0.006	0.568
	(0.838)	(0.832)	(0.814)	(0.821)
Observations	2,000	2,000	2,000	2,000
R-squared	0.457	0.421	0.472	0.451

*Notes*: This table reports regression results examining heterogeneous treatment effects by source of information. The dependent variable is subjective infection risk. The independent variables are *Traditional Media* (a dummy variable equal to one if the respondent’s primary media type is television or newspaper, and zero otherwise), the treatment group dummy, and their interaction. All specifications control for individual characteristics, prefecture fixed effects, and primary media type dummies. However, coefficients for these variables are omitted from the table for brevity. In each column, the regression sample consists of respondents in the control group and one treatment group—Tokyo, Okinawa, Expert, and Government, respectively. Robust standard errors are in parentheses. * p < 0.1, ** p < 0.05, *** p < 0.01.

Overall, statistically significant differences in the response to information treatment are relatively sparse and are not robust across alternative experimental setups. In addition, because the analyses are conducted across many subgroups, some of the statistically significant findings may arise by chance due to multiple comparisons (i.e., Type I error), so we interpret the results of heterogeneity analyses with caution.

In addition to statistical significance, it is useful to assess the quantitative significance of our estimates. We take the following approach to assessing quantitative significance. For the baseline analysis in Table 3, we compare the estimated treatment effects with the sample mean prior risk perception. For the heterogeneity analysis in Tables 4–8, we focus on statistically significant results and calculate the treatment effect for the corresponding subgroup (e.g., respondents with high prior risk perceptions or chronic diseases) by taking the sum of the coefficients on the treatment group dummy and the interaction term. We also test whether this combined effect is statistically different from zero.

Following this approach, the estimated treatment effects in the baseline analysis, which range from −0.63 to 0.45 percentage points, are relatively small compared with the mean prior risk perception of 9.65% (see Fig 2). Meanwhile, the heterogeneous treatment effects for some subgroups are quantitatively large and statistically significant, albeit marginally at the 10% level for some cases. For example, among respondents with high prior risk, the combined effect is −4.54 percentage points for the Okinawa treatment (*p* = 0.038) and −3.61 percentage points for the Government treatment (*p* = 0.091). Among respondents with chronic diseases, the combined effect is −3.02 percentage points for the Okinawa treatment (*p* = 0.026) and 2.97 percentage points for the Expert treatment (*p* = 0.064). However, as noted above, these heterogeneous effects are not robust to alternative setups, so we interpret them with caution.

In the analyses above, our dependent variable—subjective infection risk—is measured using the midpoints of the probability categories provided in the survey. Using these midpoints allows us to continue expressing infection risk in probability terms, while facilitating estimation using linear regression and a more straightforward interpretation of the treatment effects. As a sensitivity analysis, in S4 Appendix we use the original ordered probability categories and estimate our model specifications using ordered logistic regressions. The results for the average treatment effects are consistent with the baseline estimates, and the heterogeneity analyses show broadly similar patterns.

## Discussion

We find that none of the information treatments meaningfully changed respondents’ perceived infection risk. This finding is in stark contrast to [9], which conducted a similar experiment in April 2023 and found quantitatively large and statistically significant effects of the following pessimistic, qualitative infection outlook on risk perceptions. In [9], the following information treatment was provided to respondents:

*‘The number of new cases has been gradually increasing, and there is concern about the spread of infection after the holidays in May. On April 19, the expert group mentioned the possibility of a 9th wave, which would be larger than the 8th wave. Compared to the 6th and 7th waves (January-April 2022 and July-September 2022), the 8th wave (November 2022-February 2023) showed an increase in fatality rate.’*

It is interesting to note that this information treatment had statistically significant and quantitatively large effects on risk perceptions, even though the infection was low and stable at the time of the survey. According to Fig 1, the number of daily new infections was near the trough between two infection waves at the time of the survey.

There are two possible reasons for the difference between [9] and the present study. The first is that the narrative about the infection outlook in [9] was more pessimistic than the three narratives in the present study. The second is that the public’s risk perception and responsiveness to COVID-19-related information may have changed between the two study periods. Many contextual factors might have contributed to this change, including changes in population immunity, changes in severity of circulating variants, pandemic fatigue, behavioral adaptation, changing media attention, and evolving public attitudes toward COVID-19. We highlight one major event that took place between [9] and our study—the reclassification of COVID-19 from Category II to Category V on May 8, 2023—which likely played a key role affecting media attention and public attitudes. This reclassification might be a key factor underlying the change in the public’s risk perception and responsiveness to COVID-19-related information.

Our results are subject to three caveats. First, our information treatments differ along multiple dimensions. Throughout the paper, we highlighted the distinction between pessimistic and optimistic narratives in characterizing different information treatments. However, these information treatments also differ in both the content and the source of information (clinic, hospital, expert, and government). As a result, we cannot separately identify whether responses to each treatment reflect the information content, the credibility of the information source, or a combination of both. We leave such an investigation to future research.

Second, there is a potential for selection bias associated with recruitment through an online survey panel administered by a marketing company. Although we matched the age and gender distributions of our sample to those of the population census, participants in online survey panels may still differ from the general population in other characteristics. If these characteristics are associated with responsiveness to information, the treatment effects estimated in our sample may differ from those in the general population. Therefore, our findings should be interpreted with caution when generalizing to the broader population.

Third, although our overall sample size is sufficiently large (as indicated by the power analysis described in Section Methods), a larger sample would allow for more precise analyses of treatment heterogeneity across subgroups. Future research should take this issue into account when determining sample size.

## Conclusion

We examined how information provision affects the public’s perceived COVID-19 infection risk in August 2023, a few months after the Japanese government reclassified COVID-19 from Category II to Category V and officially ended the PHE. We found that none of the information treatments affected the public’s risk perceptions in a statistically significant way, in stark contrast with a similar information-provision experiment conducted right before the reclassification (see [9]). There are likely many factors explaining the difference in the results, such as the difference in the degree of pessimism in the information treatment, changes in population immunity, pandemic fatigue, and behavioral adaptation, among others. One key factor might have been the reclassification of COVID-19 from Category II to Category V on May 8, 2023, which officially marked the end of the PHE.

## Supporting information

S1 Appendix Questionnaire. Full English translation of the survey.
(PDF)

S2 Appendix Results from two alternative setups. This appendix shows the effect of information provision in the two alternative setups discussed at the end of Section Methods.
(PDF)

S3 Appendix Additional heterogeneity analyses. Additional heterogeneity analyses across individual characteristics.
(PDF)

S4 Appendix Sensitivity analysis. Sensitivity analysis using ordered logistic regression models.
(PDF)
