# Supplementary material for: COVID-19 risk perceptions after the end of the public health emergency
Source: PLoS One. 2026 Nov 10;21(11):e0348400. doi: 10.1371/journal.pone.0348400 (PMC13750598; doi:10.1371/journal.pone.0348400)
Supplement: Supplementary file 1 [file pone.0348400.s001.pdf]

Supplementary Information

S1 Appendix. Questionnaire

Introduction

This survey is an academic research project conducted by the Laboratory of Taisuke Nakata, Graduate School of Economics, The University of Tokyo, and commissioned to Cross Marketing Inc.

The purpose of this survey is to understand people's perceptions of COVID-19 risks. It includes questions about past COVID-19 infection experiences, vaccination history, pre-existing medical conditions, income, and other related factors.

All responses will be statistically processed and anonymized so that no individual can be identified. Summary data or raw data may be made public, but never in a way that allows personal identification. Your data will never be used for any purpose other than this research. Please answer each question as you think.

Estimated time to complete: 10 minutes.

Participation is voluntary. You may stop at any time by closing your browser. In that case, no incentive points will be awarded.

If you agree with the above and wish to participate, please select 'Next.' If you do not wish to participate, you may end the survey here (no incentive points will be given).

Screening Survey

Respondent criteria

- Men and women aged 20–79 who are registered panel members.
- Sampling will match national representative ratios for age and gender.

Questions

S1. Please indicate your gender. (Single Answer)

- Male
- Female

S2. Please indicate your age. (Pre-coded Data)

S3. Please indicate the prefecture where you currently live. (Pre-coded Data)

S4. After reading the above information, would you be willing to participate in this survey? (Single Answer)

- Proceed to Next (Participate in the survey)
- Stop answering (Do not participate in the survey)

Main Survey

Q1. Are you currently receiving treatment or under medical observation for any of the following conditions? Please select all that apply. (Multiple Answer)

- Malignant neoplasm (cancer)
- Cerebrovascular disease (e.g., cerebral hemorrhage, cerebral infarction)
- Respiratory disease
- Cardiovascular disease (e.g., angina, myocardial infarction)
- Digestive system disease (stomach, intestines, liver, spleen, etc.)
- Endocrine disease (e.g., diabetes)
- Kidney disease
- Blood disorders (e.g., anemia)
- None

Q2. How many times in total have you received a COVID-19 vaccine so far? (Single Answer)

- 0 times

- 1 time
- 2 times
- 3 times
- 4 times
- 5 times or more

Q3. How many times in total have you been infected with COVID-19? (Single Answer)

- 0 times
- 1 time
- 2 times
- 3 times or more

Q4. (**For respondents who answered '1 time or more' to Q3**) When you were infected, did your condition become severe enough to require a ventilator or ECMO (Extracorporeal Membrane Oxygenation)? (Single Answer)

- Became severe
- Did not become severe

Q5. Do you know anyone who died after being infected with COVID-19? (Single Answer)

- Yes
- No

Q6. To what extent did you practice infection prevention measures over the past month, such as handwashing and hand sanitizing, wearing masks, ventilation, and avoiding the 'Three Cs' (closed spaces, crowded places, and close-contact settings)? (Single Answer)

- Frequently
- Sometimes
- Rarely
- Almost never
- Never

Instructions for the Survey Company

- For 33% of respondents, skip Q7 and proceed directly to Q8.
- For 67% of respondents, ask Q7 and then proceed to Q8.

Information Provided Before Q7

- No information provided **(50% of those assigned to Question 7; 33% of all respondents**)
- In July 2022, the number of newly confirmed cases was 3,463,299 (account for 2.77% of the total population), while in October 2022, infection was relatively calm with 1,031,436 newly confirmed cases (0.83% of the total population) reported. (**50% of those assigned to Question 7; 33% of all respondents**)

Q7. What do you think is the probability (in percent) that you will become infected with COVID-19 within the next month? Please give your own assessment. (Single Answer)

- 20% or higher
- 10% or higher but less than 20%
- 5% or higher but less than 10%
- 1% or higher but less than 5%
- 0.1% or higher but less than 1%
- 0.01% or higher but less than 0.1%
- 0.001% or higher but less than 0.01%
- Less than 0.001%

Q8. Which of the following media do you rely on most as a source of information? (Single Answer)

- Television
- Newspapers
- Internet

- Social media (SNS)
- Radio
- Other

Q9. What is the highest level of education you have completed? (Single Answer)

- Junior high school or elementary school
- High school
- Junior college (including technical colleges)
- University
- Graduate school (master's degree)
- Graduate school (doctoral degree)

Q10. What was your household's total annual gross income in 2022, including bonuses? Please select one. (Single Answer)

- No income
- Under 1 million yen
- 1–2 million yen
- 2–4 million yen
- 4–6 million yen
- 6–8 million yen
- 8–10 million yen
- 10–12 million yen
- 12–14 million yen
- 14 million yen or more

Instructions for the Survey Company

At this point, there are three groups:

- 33%: Proceeded to Q8 without being asked Q7
- 33%: Asked Q7 without being provided infection statistics for July/October 2022
- 33%: Asked Question 7 and provided infection statistics for July/October 2022

Information Provided Before Q11

Information Provided to All Respondents

- COVID-19 has been reclassified to Category V Infectious Disease on May 8, 2023. At the same time, the system has been changed from recording the number of daily new infections ('notifiable disease surveillance') to recording the number of new infections in certain medical institutions ('sentinel surveillance').

  Before changing to the sentinel surveillance system, **265,404 new infections (0.21% of the total population) were reported in April 2023**. According to the weekly sentinel surveillance records after May 8, the number of new infections has been increasing after the reclassification of COVID-19 to Category V, and the number of new infections in the third week of July is approximately **4.2 times** that in the second week of May.

Additional Information (randomly assigned)

- No additional information **(20% of each group)**
- In early July, the director of a clinic in Tokyo said that 'the number of patients is on par with the peak of the eighth wave (the number of infections exceeded 20,000 cases per day in Tokyo), and an invisible collapse of the medical system has begun.' **(20% of each group)**
- A hospital in Okinawa held an emergency press conference on July 11 to announce the risk of collapse of the medical system due to a resurgence of infections, urging citizens not to relax basic preventive measures. **(20% of each group)**
- On July 16, Shigeru Omi, the head of the government's COVID-19 countermeasures subcommittee, stated that 'not only the number of new cases but also the

number of hospitalized and severe cases are increasing. This could be due to an increase in contact opportunities following the reclassification of COVID-19 and to diminished immunity from natural infections and vaccines over time. We do not know the full extent of the increase in the number of infections, but we should anticipate that this upward trend will continue'. **(20% of each group)**

- At a press conference held in early July, Shigeyuki Goto, the minister in charge of measures for novel coronavirus disease, stated that the number of patients had not increased sharply, and it was inappropriate to consider the current period as the ninth wave of infections in Japan. **(20% of each group)**

Q11. Taking into account the recent infection trends just described, please answer again: What do you think is the probability (in percent) that you will become infected with COVID-19 within the next month? Please give your own assessment. (Single Answer)

(Same options as Q7)

Q12. How much do you plan to engage in infection prevention measures over the next month, such as handwashing and hand sanitizing, wearing masks, ventilation, and avoiding the 'Three Cs'? (Single Answer)

- I plan to do so frequently
- I plan to do so sometimes
- I plan to do so rarely
- I plan to almost never do so
- I do not plan to do so at all
