# Supplementary material for: COVID-19 risk perceptions after the end of the public health emergency
Source: PLoS One. 2026 Nov 10;21(11):e0348400. doi: 10.1371/journal.pone.0348400 (PMC13750598; doi:10.1371/journal.pone.0348400)
Supplement: Supplementary file 2 [file pone.0348400.s002.pdf]

Supplementary Information

S2 Appendix. Results from two alternative setups

Table S2.1 and Table S2.2 show the effect of information provision in the two alternative setups discussed at the end of Section 2 (Survey Design). These two tables demonstrate the robustness of the key result in our paper—none of the information treatments has statistically significant effects on the public's risk perceptions.

We also conduct the heterogeneity analysis in these two alternative setups. For all of the four information treatments and for any individual attribute, we find no statistically significant heterogeneity across sub-groups. The results are not shown for the sake of brevity, but available upon request.

Table S2.1: Average effects: No anchor - prior group

	(1)
	Infection Risk
Information: Tokyo	0.725
	(0.622)
Information: Okinawa	−0.178
	(0.618)
Information: Expert	0.605
	(0.625)
Information: Government	0.317
	(0.637)
Prior Risk Above 10%	25.526***
	(0.710)
College Graduate	−1.176***
	(0.419)
High Income	−0.614
	(0.413)
Age Over 60 years	−1.006**
	(0.424)
Female	0.659
	(0.412)
Vaccination	0.352
	(0.566)
Chronic Diseases	0.097
	(0.578)
Infected with COVID-19	−0.577
	(0.497)
Acq. Died from COVID-19	0.608
	(0.846)
Constant	4.881***
	(1.212)
Observations	5,000
R-squared	0.398

Notes: This table reports regression results examining the average treatment effects of information provision. The regression sample includes respondents in the no nominal anchor and prior elicitation group. The dependent variable is subjective infection risk. The independent variables are dummy variables for information groups. Control variables include dummy variables for prior risk perception, age, gender, education, income, chronic diseases, prior infection, acquaintance's COVID-19-related death, and vaccination status. All regressions also include prefecture fixed effects and dummy variables for primary media type; however, coefficients for these variables are omitted from the table for brevity. Robust standard errors are in parentheses. * $p < 0.1$, ** $p < 0.05$, *** $p < 0.01$.

Table S2.2: Average effects: No anchor - no prior group

	(1)
	Infection Risk
Information: Tokyo	−0.788
	(0.763)
Information: Okinawa	−0.171
	(0.775)
Information: Expert	−0.044
	(0.777)
Information: Government	0.137
	(0.777)
College Graduate	−1.928***
	(0.503)
High Income	−0.527
	(0.497)
Age Over 60 years	−1.981***
	(0.548)
Female	1.335***
	(0.501)
Vaccination	2.062***
	(0.703)
Chronic Diseases	−0.560
	(0.669)
Infected with COVID-19	2.454***
	(0.622)
Acq. Died from COVID-19	2.965***
	(1.064)
Constant	9.210***
	(1.393)
Observations	5,000
R-squared	0.030

Notes: This table reports regression results examining the average treatment effects of information provision. The regression sample includes respondents in the no nominal anchor and no prior elicitation group. The dependent variable is subjective infection risk. The independent variables are dummy variables for information groups. Control variables include dummy variables for age, gender, education, income, chronic diseases, prior infection, acquaintance's COVID-19-related death, and vaccination status. All regressions also include prefecture fixed effects and dummy variables for primary media type; however, coefficients for these variables are omitted from the table for brevity. Robust standard errors are in parentheses. * $p < 0.1$, ** $p < 0.05$, *** $p < 0.01$.
