# Supplementary material for: COVID-19 risk perceptions after the end of the public health emergency
Source: PLoS One. 2026 Nov 10;21(11):e0348400. doi: 10.1371/journal.pone.0348400 (PMC13750598; doi:10.1371/journal.pone.0348400)
Supplement: Supplementary file 3 [file pone.0348400.s003.pdf]

Supplementary Information

S3 Appendix. Additional heterogeneity analyses

Table S3.1: Heterogeneity - Education level

	(1)	(2)	(3)	(4)
GROUP	Control & Tokyo	Control & Okinawa	Control & Expert	Control & Gov.
VARIABLES	Posterior Risk	Posterior Risk	Posterior Risk	Posterior Risk
College Grad.	−0.638	−0.445	−0.395	−0.952
	(0.787)	(0.786)	(0.788)	(0.787)
Treatment*College Grad.	0.649	−0.264	1.014	1.350
	(1.117)	(1.088)	(1.104)	(1.080)
Treatment dummy	0.116	−0.189	−0.138	−1.313*
	(0.811)	(0.817)	(0.795)	(0.780)
Observations	2,000	2,000	2,000	2,000
R-squared	0.457	0.421	0.472	0.451

Notes: This table reports regression results examining heterogeneous treatment effects by education level. The dependent variable is subjective infection risk. The independent variables are *College Graduates*, the treatment group dummy, and their interaction. All specifications control for individual characteristics, prefecture fixed effects, and primary media type dummies. However, coefficients for these variables are omitted from the table for brevity. In each column, the regression sample consists of respondents in the control group and one treatment group—Tokyo, Okinawa, Expert, and Government, respectively. Robust standard errors are in parentheses. * $p < 0.1$, ** $p < 0.05$, *** $p < 0.01$.

Table S3.2: Heterogeneity - Income

	(1)	(2)	(3)	(4)
GROUP	Control & Tokyo	Control & Okinawa	Control & Expert	Control & Gov.
VARIABLES	Posterior Risk	Posterior Risk	Posterior Risk	Posterior Risk
High Income	−0.618	−0.478	−0.385	−0.683
	(0.779)	(0.783)	(0.780)	(0.781)
Treatment*High Income	0.493	−0.155	1.004	0.544
	(1.127)	(1.082)	(1.119)	(1.075)
Treatment dummy	0.177	−0.237	−0.161	−0.935
	(0.816)	(0.831)	(0.787)	(0.772)
Observations	2,000	2,000	2,000	2,000
R-squared	0.457	0.421	0.472	0.450

Notes: This table reports regression results examining heterogeneous treatment effects by income. The dependent variable is subjective infection risk. The independent variables are *High Income*, the treatment group dummy, and their interaction. All specifications control for individual characteristics, prefecture fixed effects, and primary media type dummies. However, coefficients for these variables are omitted from the table for brevity. In each column, the regression sample consists of respondents in the control group and one treatment group—Tokyo, Okinawa, Expert, and Government, respectively. Robust standard errors are in parentheses. * $p < 0.1$, ** $p < 0.05$, *** $p < 0.01$.

Table S3.3: Heterogeneity - Vaccination

GROUP<br>VARIABLES	(1)<br>Control & Tokyo<br>Posterior Risk	(2)<br>Control & Okinawa<br>Posterior Risk	(3)<br>Control & Expert<br>Posterior Risk	(4)<br>Control & Gov.<br>Posterior Risk
Vaccination	0.817	0.878	0.033	0.694
	(1.068)	(1.110)	(1.062)	(1.066)
Treatment*Vaccination	0.510	−0.594	−1.463	−0.505
	(1.441)	(1.492)	(1.562)	(1.466)
Treatment dummy	−0.003	0.188	1.575	−0.233
	(1.314)	(1.387)	(1.430)	(1.342)
Observations	2,000	2,000	2,000	2,000
R-squared	0.457	0.421	0.472	0.450

Notes: This table reports regression results examining heterogeneous treatment effects by vaccination status. The dependent variable is subjective infection risk. The independent variables are *Vaccination*, the treatment group dummy, and their interaction. All specifications control for individual characteristics, prefecture fixed effects, and primary media type dummies. However, coefficients for these variables are omitted from the table for brevity. In each column, the regression sample consists of respondents in the control group and one treatment group—Tokyo, Okinawa, Expert, and Government, respectively. Robust standard errors are in parentheses. * $p < 0.1$, ** $p < 0.05$, *** $p < 0.01$.

Table S3.4: Heterogeneity - Infection

GROUP<br>VARIABLES	(1)<br>Control & Tokyo<br>Posterior Risk	(2)<br>Control & Okinawa<br>Posterior Risk	(3)<br>Control & Expert<br>Posterior Risk	(4)<br>Control & Gov.<br>Posterior Risk
Infected with COVID-19	−0.761	−0.472	−0.616	−0.671
	(0.926)	(0.921)	(0.926)	(0.913)
Treatment*Infected with	0.995	1.118	0.201	0.201
COVID-19	(1.429)	(1.305)	(1.401)	(1.401)
Treatment dummy	0.186	−0.591	0.298	0.298
	(0.624)	(0.639)	(0.625)	(0.625)
Observations	2,000	2,000	2,000	2,000
R-squared	0.457	0.421	0.472	0.450

Notes: This table reports regression results examining heterogeneous treatment effects by past COVID-19 infection experiences. The dependent variable is subjective infection risk. The independent variables are *Infected with COVID-19*, the treatment group dummy, and their interaction. All specifications control for individual characteristics, prefecture fixed effects, and primary media type dummies. However, coefficients for these variables are omitted from the table for brevity. In each column, the regression sample consists of respondents in the control group and one treatment group—Tokyo, Okinawa, Expert, and Government, respectively. Robust standard errors are in parentheses. * $p < 0.1$, ** $p < 0.05$, *** $p < 0.01$.
