# Supplementary material for: COVID-19 risk perceptions after the end of the public health emergency
Source: PLoS One. 2026 Nov 10;21(11):e0348400. doi: 10.1371/journal.pone.0348400 (PMC13750598; doi:10.1371/journal.pone.0348400)
Supplement: Supplementary file 4 [file pone.0348400.s004.pdf]

Supplementary Information

S4 Appendix. Sensitivity analysis

Table S4.1: Average effects: Ordered logistic regression

	(1)
	Infection Risk
Information: Tokyo	0.050
	(0.081)
Information: Okinawa	−0.023
	(0.079)
Information: Expert	0.058
	(0.080)
Information: Government	−0.041
	(0.079)
Prior Risk Above 10%	3.836***
	(0.101)
College Graduate	0.072
	(0.055)
High Income	0.057
	(0.053)
Age Over 60 years	−0.147***
	(0.056)
Female	0.141***
	(0.054)
Vaccination	0.707***
	(0.084)
Chronic Diseases	0.121*
	(0.070)
Infected with COVID-19	0.207***
	(0.062)
Acq. Died from COVID-19	0.170
	(0.103)
Observations	5,000

Notes: This table reports ordered logistic regression results examining the average treatment effects of information provision. The regression sample includes respondents in the nominal anchor and prior elicitation group. The dependent variable is subjective infection risk, measured as an ordinal variable ranging from 1 (less than 0.001%) to 8 (20% or higher). The independent variables are dummy variables for information groups. Control variables include dummy variables for age, gender, education, income, chronic diseases, prior infection, acquaintance's COVID-19-related death, and vaccination status. All regressions also include prefecture fixed effects and dummy variables for primary media type; however, coefficients for these variables are omitted from the table for brevity. Robust standard errors are in parentheses. * $p < 0.1$, ** $p < 0.05$, *** $p < 0.01$.

Table S4.2: Heterogeneity - Prior risk (Ordered logistic regression)

	(1)	(2)	(3)	(4)
GROUP	Control & Tokyo	Control & Okinawa	Control & Expert	Control & Gov.
VARIABLES	Infection Risk	Infection Risk	Infection Risk	Infection Risk
Prior Risk Above 10%	4.161***	4.213***	4.280***	4.332***
	(0.183)	(0.186)	(0.193)	(0.196)
Treatment*Prior Risk	−0.217	−0.535**	−0.121	−0.366*
Above 10%	(0.217)	(0.219)	(0.214)	(0.218)
Treatment dummy	0.089	0.079	0.081	0.041
	(0.092)	(0.091)	(0.092)	(0.091)
Observations	2,000	2,000	2,000	2,000

Notes: This table reports ordered logistic regression results examining heterogeneous treatment effects by prior infection risk. The regression sample includes respondents in the nominal anchor and prior elicitation group. The dependent variable is subjective infection risk, measured as an ordinal variable ranging from 1 (less than 0.001%) to 8 (20% or higher). The independent variables are *Prior Risk Above 10%*, the treatment group dummy, and their interaction. All specifications control for individual characteristics, prefecture fixed effects, and primary media type dummies. However, coefficients for these variables are omitted from the table for brevity. In each column, the regression sample consists of respondents in the control group and one treatment group—Tokyo, Okinawa, Expert, and Government, respectively. Robust standard errors are in parentheses. * $p < 0.1$, ** $p < 0.05$, *** $p < 0.01$.

Table S4.3: Heterogeneity - Education level (Ordered logistic regression)

	(1)	(2)	(3)	(4)
GROUP	Control & Tokyo	Control & Okinawa	Control & Expert	Control & Gov.
VARIABLES	Infection Risk	Infection Risk	Infection Risk	Infection Risk
College Grad.	0.085	0.078	0.069	0.024
	(0.121)	(0.123)	(0.122)	(0.124)
Treatment*College Grad.	0.035	−0.146	−0.022	0.099
	(0.168)	(0.167)	(0.168)	(0.168)
Treatment dummy	0.028	0.047	0.066	−0.082
	(0.123)	(0.123)	(0.120)	(0.120)
Observations	2,000	2,000	2,000	2,000

Notes: This table reports ordered logistic regression results examining heterogeneous treatment effects by education level. The regression sample includes respondents in the nominal anchor and prior elicitation group. The dependent variable is subjective infection risk, measured as an ordinal variable ranging from 1 (less than 0.001%) to 8 (20% or higher). The independent variables are *College Graduates*, the treatment group dummy, and their interaction. All specifications control for individual characteristics, prefecture fixed effects, and primary media type dummies. However, coefficients for these variables are omitted from the table for brevity. In each column, the regression sample consists of respondents in the control group and one treatment group—Tokyo, Okinawa, Expert, and Government, respectively. Robust standard errors are in parentheses. * $p < 0.1$, ** $p < 0.05$, *** $p < 0.01$.

Table S4.4: Heterogeneity - Income (Ordered logistic regression)

	(1)	(2)	(3)	(4)
GROUP	Control & Tokyo	Control & Okinawa	Control & Expert	Control & Gov.
VARIABLES	Infection Risk	Infection Risk	Infection Risk	Infection Risk
High Income	0.111	0.138	0.157	0.113
	(0.115)	(0.118)	(0.116)	(0.118)
Treatment*High Income	−0.316*	−0.191	0.000	0.082
	(0.168)	(0.166)	(0.168)	(0.168)
Treatment dummy	0.210*	0.075	0.055	−0.075
	(0.123)	(0.126)	(0.124)	(0.121)
Observations	2,000	2,000	2,000	2,000

Notes: This table reports ordered logistic regression results examining heterogeneous treatment effects by income. The regression sample includes respondents in the nominal anchor and prior elicitation group. The dependent variable is subjective infection risk, measured as an ordinal variable ranging from 1 (less than 0.001%) to 8 (20% or higher). The independent variables are *High Income*, the treatment group dummy, and their interaction. All specifications control for individual characteristics, prefecture fixed effects, and primary media type dummies. However, coefficients for these variables are omitted from the table for brevity. In each column, the regression sample consists of respondents in the control group and one treatment group—Tokyo, Okinawa, Expert, and Government, respectively. Robust standard errors are in parentheses. * p < 0.1, ** p < 0.05, *** p < 0.01.

Table S4.5: Heterogeneity - Age (Ordered logistic regression)

	(1)	(2)	(3)	(4)
GROUP	Control & Tokyo	Control & Okinawa	Control & Expert	Control & Gov.
VARIABLES	Infection Risk	Infection Risk	Infection Risk	Infection Risk
Age Over 60 years	−0.279**	−0.295**	−0.320***	−0.271**
	(0.120)	(0.123)	(0.121)	(0.122)
Treatment*Age Over 60 years	0.032	0.134	0.364**	0.144
	(0.168)	(0.170)	(0.170)	(0.168)
Treatment dummy	0.034	−0.073	−0.074	−0.086
	(0.107)	(0.105)	(0.105)	(0.109)
Observations	2,000	2,000	2,000	2,000

Notes: This table reports ordered logistic regression results examining heterogeneous treatment effects by age. The regression sample includes respondents in the nominal anchor and prior elicitation group. The dependent variable is subjective infection risk, measured as an ordinal variable ranging from 1 (less than 0.001%) to 8 (20% or higher). The independent variables are *Age Over 60 years*, the treatment group dummy, and their interaction. All specifications control for individual characteristics, prefecture fixed effects, and primary media type dummies. However, coefficients for these variables are omitted from the table for brevity. In each column, the regression sample consists of respondents in the control group and one treatment group—Tokyo, Okinawa, Expert, and Government, respectively. Robust standard errors are in parentheses. * p < 0.1, ** p < 0.05, *** p < 0.01.

Table S4.6: Heterogeneity - Gender (Ordered logistic regression)

GROUP<br>VARIABLES	(1)<br>Control & Tokyo<br>Infection Risk	(2)<br>Control & Okinawa<br>Infection Risk	(3)<br>Control & Expert<br>Infection Risk	(4)<br>Control & Gov.<br>Infection Risk
Female	0.088	0.033	0.081	0.087
	(0.118)	(0.121)	(0.118)	(0.121)
Treatment*Female	0.147	0.159	0.149	−0.087
	(0.168)	(0.165)	(0.166)	(0.167)
Treatment dummy	−0.028	−0.106	−0.019	0.010
	(0.116)	(0.117)	(0.115)	(0.116)
Observations	2,000	2,000	2,000	2,000

Notes: This table reports ordered logistic regression results examining heterogeneous treatment effects by gender. The regression sample includes respondents in the nominal anchor and prior elicitation group. The dependent variable is subjective infection risk, measured as an ordinal variable ranging from 1 (less than 0.001%) to 8 (20% or higher). The independent variables are *Female*, the treatment group dummy, and their interaction. All specifications control for individual characteristics, prefecture fixed effects, and primary media type dummies. However, coefficients for these variables are omitted from the table for brevity. In each column, the regression sample consists of respondents in the control group and one treatment group—Tokyo, Okinawa, Expert, and Government, respectively. Robust standard errors are in parentheses. * $p < 0.1$, ** $p < 0.05$, *** $p < 0.01$.

Table S4.7: Heterogeneity - Vaccination (Ordered logistic regression)

GROUP<br>VARIABLES	(1)<br>Control & Tokyo<br>Infection Risk	(2)<br>Control & Okinawa<br>Infection Risk	(3)<br>Control & Expert<br>Infection Risk	(4)<br>Control & Gov.<br>Infection Risk
Vaccination	0.765***	0.820***	0.725***	0.790***
	(0.173)	(0.179)	(0.171)	(0.183)
Treatment*Vaccination	−0.004	−0.166	−0.220	0.161
	(0.254)	(0.253)	(0.262)	(0.259)
Treatment dummy	0.048	0.116	0.243	−0.172
	(0.239)	(0.238)	(0.248)	(0.242)
Observations	2,000	2,000	2,000	2,000

Notes: This table reports ordered logistic regression results examining heterogeneous treatment effects by vaccination status. The regression sample includes respondents in the nominal anchor and prior elicitation group. The dependent variable is subjective infection risk, measured as an ordinal variable ranging from 1 (less than 0.001%) to 8 (20% or higher). The independent variables are *Vaccination*, the treatment group dummy, and their interaction. All specifications control for individual characteristics, prefecture fixed effects, and primary media type dummies. However, coefficients for these variables are omitted from the table for brevity. In each column, the regression sample consists of respondents in the control group and one treatment group—Tokyo, Okinawa, Expert, and Government, respectively. Robust standard errors are in parentheses. * $p < 0.1$, ** $p < 0.05$, *** $p < 0.01$.

Table S4.8: Heterogeneity - Health (Ordered logistic regression)

GROUP<br>VARIABLES	(1)<br>Control & Tokyo<br>Infection Risk	(2)<br>Control & Okinawa<br>Infection Risk	(3)<br>Control & Expert<br>Infection Risk	(4)<br>Control & Gov.<br>Infection Risk
Chronic Diseases	0.249	0.242	0.200	0.232
	(0.157)	(0.157)	(0.154)	(0.158)
Treatment*Chronic	−0.249	−0.392*	0.203	−0.132
Diseases	(0.233)	(0.230)	(0.215)	(0.221)
Treatment dummy	0.081	0.032	0.026	−0.015
	(0.092)	(0.091)	(0.091)	(0.091)
Observations	2,000	2,000	2,000	2,000

Notes: This table reports ordered logistic regression results examining heterogeneous treatment effects by health status. The regression sample includes respondents in the nominal anchor and prior elicitation group. The dependent variable is subjective infection risk, measured as an ordinal variable ranging from 1 (less than 0.001%) to 8 (20% or higher). The independent variables are *Chronic Diseases*, the treatment group dummy, and their interaction. All specifications control for individual characteristics, prefecture fixed effects, and primary media type dummies. However, coefficients for these variables are omitted from the table for brevity. In each column, the regression sample consists of respondents in the control group and one treatment group—Tokyo, Okinawa, Expert, and Government, respectively. Robust standard errors are in parentheses. * p < 0.1, ** p < 0.05, *** p < 0.01.

Table S4.9: Heterogeneity - Infection (Ordered logistic regression)

GROUP<br>VARIABLES	(1)<br>Control & Tokyo<br>Infection Risk	(2)<br>Control & Okinawa<br>Infection Risk	(3)<br>Control & Expert<br>Infection Risk	(4)<br>Control & Gov.<br>Infection Risk
Infected with COVID-19	0.238*	0.264**	0.247*	0.236*
	(0.130)	(0.133)	(0.133)	(0.133)
Treatment*Infected with	−0.038	0.128	−0.240	−0.116
COVID-19	(0.197)	(0.196)	(0.200)	(0.193)
Treatment dummy	0.054	−0.057	0.111	−0.005
	(0.095)	(0.095)	(0.094)	(0.096)
Observations	2,000	2,000	2,000	2,000

Notes: This table reports ordered logistic regression results examining heterogeneous treatment effects by past COVID-19 infection experiences. The regression sample includes respondents in the nominal anchor and prior elicitation group. The dependent variable is subjective infection risk, measured as an ordinal variable ranging from 1 (less than 0.001%) to 8 (20% or higher). The independent variables are *Infected with COVID-19*, the treatment group dummy, and their interaction. All specifications control for individual characteristics, prefecture fixed effects, and primary media type dummies. However, coefficients for these variables are omitted from the table for brevity. In each column, the regression sample consists of respondents in the control group and one treatment group—Tokyo, Okinawa, Expert, and Government, respectively. Robust standard errors are in parentheses. * p < 0.1, ** p < 0.05, *** p < 0.01.

Table S4.10: Heterogeneity - Acquaintance death (Ordered logistic regression)

GROUP<br>VARIABLES	(1)<br>Control & Tokyo<br>Infection Risk	(2)<br>Control & Okinawa<br>Infection Risk	(3)<br>Control & Expert<br>Infection Risk	(4)<br>Control & Gov.<br>Infection Risk
Acq. Died	0.495**	0.456**	0.511**	0.465**
from COVID-19	(0.203)	(0.211)	(0.200)	(0.207)
Treatment*Acq. Died	−0.305	0.008	−0.540	−0.746**
from COVID-19	(0.321)	(0.317)	(0.343)	(0.311)
Treatment dummy	0.065	−0.026	0.093	0.027
	(0.087)	(0.086)	(0.086)	(0.087)
Observations	2,000	2,000	2,000	2,000

Notes: This table reports ordered logistic regression results examining heterogeneous treatment effects by COVID-19-related acquaintance death. The regression sample includes respondents in the nominal anchor and prior elicitation group. The dependent variable is subjective infection risk, measured as an ordinal variable ranging from 1 (less than 0.001%) to 8 (20% or higher). The independent variables are *Acq. Died from COVID-19*, the treatment group dummy, and their interaction. All specifications control for individual characteristics, prefecture fixed effects, and primary media type dummies. However, coefficients for these variables are omitted from the table for brevity. In each column, the regression sample consists of respondents in the control group and one treatment group—Tokyo, Okinawa, Expert, and Government, respectively. Robust standard errors are in parentheses. * $p < 0.1$, ** $p < 0.05$, *** $p < 0.01$.

Table S4.11: Heterogeneity - Population density (Ordered logistic regression)

GROUP<br>VARIABLES	(1)<br>Control & Tokyo<br>Infection Risk	(2)<br>Control & Okinawa<br>Infection Risk	(3)<br>Control & Expert<br>Infection Risk	(4)<br>Control & Gov.<br>Infection Risk
High Density	0.511	0.754	−0.193	1.033*
	(0.675)	(0.564)	(0.610)	(0.553)
Treatment*High Density	0.140	−0.145	0.315	0.144
	(0.212)	(0.211)	(0.214)	(0.214)
Treatment dummy	−0.067	0.089	−0.198	−0.148
	(0.190)	(0.189)	(0.193)	(0.193)
Observations	2,000	2,000	2,000	2,000

Notes: This table reports ordered logistic regression results examining heterogeneous treatment effects by population density. The regression sample includes respondents in the nominal anchor and prior elicitation group. The dependent variable is subjective infection risk, measured as an ordinal variable ranging from 1 (less than 0.001%) to 8 (20% or higher). The independent variables are *High Density* (a dummy variable equal to one if the respondent resides in a prefecture with above-median population density, and zero otherwise), the treatment group dummy, and their interaction. Prefecture-level population density used in this analysis is obtained from the 2020 Population Census. All specifications control for individual characteristics, prefecture fixed effects, and primary media type dummies. However, coefficients for these variables are omitted from the table for brevity. In each column, the regression sample consists of respondents in the control group and one treatment group—Tokyo, Okinawa, Expert, and Government, respectively. Robust standard errors are in parentheses. * $p < 0.1$, ** $p < 0.05$, *** $p < 0.01$.

Table S4.12: Heterogeneity - Source of information (Ordered logistic regression)

	(1)	(2)	(3)	(4)
GROUP	Control & Tokyo	Control & Okinawa	Control & Expert	Control & Gov.
VARIABLES	Infection Risk	Infection Risk	Infection Risk	Infection Risk
Traditional Media	0.395	0.506	0.228	0.376
	(0.367)	(0.361)	(0.316)	(0.326)
Treatment*Traditional	0.085	0.043	0.308*	0.014
Media	(0.167)	(0.170)	(0.166)	(0.169)
Treatment dummy	−0.003	−0.050	−0.115	−0.041
	(0.129)	(0.128)	(0.124)	(0.126)
Observations	2,000	2,000	2,000	2,000

Notes: This table reports ordered logistic regression results examining heterogeneous treatment effects by source of information. The regression sample includes respondents in the nominal anchor and prior elicitation group. The dependent variable is subjective infection risk, measured as an ordinal variable ranging from 1 (less than 0.001%) to 8 (20% or higher). The independent variables are *Traditional Media* (a dummy variable equal to one if the respondent's primary media type is television or newspaper, and zero otherwise), the treatment group dummy, and their interaction. All specifications control for individual characteristics, prefecture fixed effects, and primary media type dummies. However, coefficients for these variables are omitted from the table for brevity. In each column, the regression sample consists of respondents in the control group and one treatment group—Tokyo, Okinawa, Expert, and Government, respectively. Robust standard errors are in parentheses. * $p < 0.1$, ** $p < 0.05$, *** $p < 0.01$.
